# Molecular epidemiological surveillance of Africa and Asia imported malaria in Wuhan, Central China: comparison of diagnostic tools during 2011–2018

**DOI:** 10.1186/s12936-020-03387-2

**Published:** 2020-09-03

**Authors:** Yiting Xie, Kai Wu, Weijia Cheng, Tingting Jiang, Yi Yao, Mingxing Xu, Yan Yang, Huabing Tan, Jian Li

**Affiliations:** 1 Department of Human Parasitology, School of Basic Medical Sciences, Hubei University of Medicine, Shiyan, 442000 People’s Republic of China; 2 Department of Infectious Diseases, Renmin Hospital, Hubei University of Medicine, Shiyan, 442000 People’s Republic of China; 3 Department of Schistosomiasis and Endemic Diseases, Wuhan City Center for Disease Prevention and Control, Wuhan, 430015 People’s Republic of China

**Keywords:** Imported malaria, Microscopic examination, Molecular diagnosis, Rapid diagnosis test, Nested PCR, Real-time PCR

## Abstract

**Background:**

Malaria remains a serious public health problem globally. As the elimination of indigenous malaria continues in China, imported malaria has gradually become a major health hazard. Well-timed and accurate diagnoses could support the timely implementation of therapeutic schedules, reveal the prevalence of imported malaria and avoid transmission of the disease.

**Methods:**

Blood samples were collected in Wuhan, China, from August 2011 to December 2018. All patients accepted microscopy and rapid diagnosis test (RDT) examinations. Subsequently, each of the positive or suspected positive cases was tested for four human-infectious *Plasmodium* species by using 18S rRNA-based nested PCR and Taqman probe-based real-time PCR. The results of the microscopy and the two molecular diagnostic methods were analysed. Importation origins were traced by country, and the prevalence of *Plasmodium* species was analysed by year.

**Results:**

A total of 296 blood samples, including 288 that were microscopy and RDT positive, 7 RDT and *Plasmodium falciparum* positive, and 1 suspected case, were collected and reanalysed. After application of the two molecular methods and sequencing, 291 cases including 245 *P. falciparum*, 15 *Plasmodium vivax*, 20 *Plasmodium ovale*, 6 *Plasmodium malariae* and 5 mixed infections (3 *P. falciparum* +* P. ovale*, 2 *P. vivax *+ *P. ovale*) were confirmed. These patients had returned from Africa (95.53%) and Asia (4.47%). Although the prevalence displayed a small-scale fluctuation, the overall trend of the imported cases increased yearly.

**Conclusions:**

These results emphasize the necessity of combined utilization of the four tools for malaria diagnosis in clinic and in field surveys of potential risk regions worldwide including Wuhan.

## Background

Malaria remains a serious threat to public health around the world, with an estimated 228 million cases and 405,000 deaths in 2018 worldwide [1]. The main epidemic areas of malaria are distributed in Africa (93%), followed by Southeast Asia (SE Asia) (3.4%) and the Eastern Mediterranean Region (2.1%) [1]. The World Health Organization (WHO) hopes to eliminate malaria in at least 35 additional countries (based on data from 2015) by 2030 [2]. To assist in this goal, China planned to eliminate the disease by 2020 [3]. In China, the occurrence and burden of indigenous malaria have rapidly decreased due to an integrated malaria control and elimination strategy implemented in 2000 [4]. It is worth noting that as of 2017, no cases of indigenous malaria have been reported in the entire country. In accordance with the nationwide status, the control of indigenous malaria in the Hubei Province of China was successful, and zero indigenous malaria cases have been reported in Hubei since 2013 [5]. However, imported malaria has gradually become a major threat in need of a process of prevention, control and elimination in endemic and non-endemic areas globally, including in China. Furthermore, it is worth mentioning that imported malaria cases from Africa and SE Asia have been gradually increasing yearly in China, including in the provincial capital of the Hubei province, Wuhan [4, 5]. For efficient control and in order to achieve malaria eradication before 2020 in China according to the state plan [3], imported malaria patients in China, especially in first-tier cities with a high population density and mobility, such as Wuhan, need to be quickly noticed.

There are five primary *Plasmodium* species causing malaria in humans, including *Plasmodium falciparum*, *Plasmodium vivax*, *Plasmodium ovale*, *Plasmodium malariae*, and *Plasmodium knowlesi*. Different *Plasmodium* species infections can cause dissimilar disease characteristics. Amongst them, *P. falciparum* is responsible for most of the morbidity and mortality of humans in sub-Saharan Africa, and other malarious tropical areas around the world [6, 7]. Populations such as young children, older adults, immunosuppressed patients and travellers from malaria-free zones to epidemic areas are particularly susceptible to falciparum malaria. *Plasmodium falciparum* can also increase the risk of severe malaria and adverse complications in pregnant women, such as maternal death, miscarriage, stillbirth and neonatal death. If patients fail to get diagnosis and treated within 24 h after the onset of falciparum malaria, it may be fatal [8]. *Plasmodium vivax* is generally the culprit for the majority of malaria cases in areas with tropical and temperate climates and is considered to be a neglected tropical disease [9, 10]. Severe *P. vivax* malaria cases have been reported in endemic areas of (sub) tropical countries. For *P. vivax*, a dormant form named hypnozoite, could stay in the liver and may lead to the malaria relapses several months or even years later [8]. *Plasmodium ovale* is mainly prevalent in Africa and Asia, particularly in West Africa [7]. *Plasmodium ovale* seldom leads to severe malaria among people in endemic regions, but can cause severe malaria disease in naive visitors [11, 12]. It is comprised of two subspecies, *P. ovale curtisi* and *P. ovale wallikeri* [13]. *Plasmodium ovale curtisi* has a significantly longer latency duration than *P. o. wallikeri* [14, 15]. Like *P. vivax*, *P. ovale* can also form hypnozoites in the host liver, which makes the prevention and treatment of both vivax and ovale malaria more difficult [8]. *Plasmodium malariae* cases have been reported worldwide and are particularly prevalent in West Africa. *Plasmodium malariae* usually causes the mildest infections and very rarely has life-threatening results. However, it may cause splenomegaly or renal damage during chronic infection [7]. *Plasmodium knowlesi* was initially found in SE Asia in wild macaques, but it can also infect humans [16]. Although human-mosquito-human transmission has not been reported for this malaria species, it is a health threat to people in forested areas of SE Asia because it may cause organ failure or even fatal outcomes [8, 17, 18]. Beyond that, simultaneous infections with more than one human *Plasmodium* species commonly happen, especially in endemic regions [19]. However, the influence of malaria caused by *P. vivax*, *P. ovale* and *P. malariae* as well as mixed infections is frequently underestimated [12, 15, 20–22]. Most importantly, different *Plasmodium* infections should be treated with the corresponding chemoprophylaxis and appropriate treatment principles [8]. Therefore, for timely and efficient treatment and to prevent malaria transmission, early and accurate diagnosis of *Plasmodium* species infection is meaningful.

As it is inexpensive and has a relatively high sensitivity for species recognition and even parasite density quantification, microscopic examination of thick and thin blood smear is traditionally regarded as the gold standard for malaria detection [23, 24]. However, the use of microscopic examination has been restricted because it requires well-trained laboratory personnel and shows less sensitive for low parasitaemia levels [25–27]. Besides, different observers may have two to three-fold discrepancies in parasite quantification [28]. More importantly, mixed infections of different *Plasmodium* species are frequently missed [29]. The two subspecies of *P. ovale* (*P. o. curtisi* and *P. o. wallikeri*) cannot be distinguished by microscopy. Misidentification may also happen between different species [30]. All of the above may induce the unreasonable usage of anti-malarial drugs, hamper parasite clearance and lead to the transmission of malaria, and even anti-malarial drug resistance. The rapid diagnosis test (RDT) as an immunologic method usually targets *Plasmodium*-specific antigens in blood samples such as histidine-rich protein 2 (HRP2) and lactate dehydrogenase (LDH) [7]. RDT offers a rapid diagnosis and can be conducted by users without extensive training. It often has comparable sensitivity to microscopy. However, RDT can only distinguish falciparum and non-falciparum *Plasmodium* species infection [31]. Furthermore, false negative results by RDT were also reported in previous studies [32–34]. Therefore, molecular tools such as nested PCR and real-time PCR that could not only detect *Plasmodium* infection, but also allow for accurate species identification by primer design should be used. Nested PCR with two rounds of amplification can improve the detection sensitivity. Sequencing of products from nested PCR represents a further reliable guarantee for species identification [35, 36]. The major advantages of real-time PCR include direct result reading without the need for downstream analysis or quantification of the DNA copy number [37, 38].

Using suitable and effective methods to timely identify imported malaria is essential for successfully completing the target assessment task for malaria elimination in China, Africa and SE Asia. Therefore, the present study aimed to confirm the *Plasmodium* species involved in all imported malaria cases in Wuhan by using microscopy, RDT, nested PCR and real-time PCR. The performances of these methods in malaria diagnosis were assessed to supply more reference values for their future use. Analysis of their diagnostic results revealed the prevalence and characteristics of imported malaria in Wuhan, China.

## Methods

### Collection of study specimens

Blood samples from clinically suspected patients with symptoms of malaria were cumulatively collected from the Center for Disease Prevention and Control (CDC) of Wuhan, China, from August 2011 to December 2018. Approximately 2 to 5 ml blood was drawn from each patient for microscopy and RDT testing and 400 μl of each sample was stored on 3 MM Whatman filter paper for molecular verification. Ethical approval for this study was obtained from the Medical Ethics Committee of the Hubei University of Medicine and Wuhan CDC. Informed consent was provided by all participating individuals.

### Microscopic examination and RDT assays

The blood samples were first subjected to One Step Malaria HRP2/pLDH (P.f/Pan) (Wondfo, Guangzhou, China) detection. Subsequently, thick and thin peripheral blood smears were made by the standard method [39]. Giemsa staining and microscopic examinations were conducted by professionals in the Wuhan CDC. Parasitaemia (parasites/μl) was determined according to the previous report [40]. No asexual form of *Plasmodium* detected in 200 high-power fields in the thin blood films and no parasites for every 1000 white blood cells were considered as negative [39, 41].

### Genomic DNA extraction

Genomic DNA (gDNA) from the microscopic-positive or suspected positive blood samples was extracted using a TIANamp blood DNA kit (Tiangen Biotech Co., Ltd., Beijing, China). Briefly, 6 mm × 6 mm blood spot (approximately 130 μl peripheral blood) was treated following the manufacturer’s instruction and finally dissolved in 50 μl elution buffer. After quantification with Gene 5, the purified gDNA was packed as aliquots and stored at − 20 °C until further use.

### Taqman probe based Real-time PCR

Probes were designed for the parasite species, including *P. falciparum*, *P. vivax*, *P. ovale* (*P. o. curtisi*, *P. o. wallikeri*) and *P. malariae*, so they could be distinguished with real-time PCR. The preparation of primers and Taqman probes was according to previous documents [35, 42] (the primers are listed in Table 1). Reactions were performed using the Premix EX Taq ™ probe qPCR (Takara, Japan) following the manufacturer’s instructions. Briefly, a reaction mixture consisted of 12.5 μl premix Ex Taq (2×), 0.5 μl of each primer (final 0.2 μM), and approximately 1 μl fluorescence probe (final 0.1–0.5 μM), and 2 μl DNA template in a final total volume of 25 μl supplemented with ultrapure water. All reactions were performed as suggested by the manufacturer, including 1 cycle of 95 °C for 30 s, 40 repeated cycles of 95 °C for 5 s and 60 °C for 30 s, on a CFX96 real-time PCR machine (Bio-Rad, USA).

**Table 1:** Primers and probes for *Plasmodium* species detection

Method	Species	Primer	Sequence (5′–3′)	Length (bp)	References
Nested PCR	*Plasmodium* sp.	rPLU1	TCAAAGATTAAGCCATGCAAGTGA	1670	[43]
		rPLU5	CCTGTTGTTGCCTTAAACTTC		
	*P. falciparum*	rFAL1	TTAAACTGGTTTGGGAAAACCAAATATATT	205	
		rFAL2	ACACAATGAACTCAATCATGACTACCCGTC		
	*P. vivax*	rPVIV1	CGCTTCTAGCTTAATCCACATAACTGATAC	121	
		rPVIV2	ACTTCCAAGCCGAAGCAAAGAAAGTCCTTA		
	*P. malariae*	rMAL1	ATAACATAGTTGTACGTTAAGAATAACCGC	145	
		rMAL2	AAAATTCCCATGCATAAAAAATTATACAAA		
	*P. ovale*	rOVA1WC	TGTAGTATTCAAACGCAGT	659–662	
		rOVA2WC	TATGTACTTGTTAAGCCTTT		
	*P. ovale curtisi*	rOVA1	ATCTCTTTTGCTATTTTTTAGTATTGGAGA	800	[44]
		rOVA2	GGAAAAGGACACATTAATTGTATCCTAGTG		
	*P. ovale wallikeri*	rOVA1v	ATCTCCTTTACTTTTTGTACTGGAGA	780	
		rOVA2v	GGAAAAGGACACTATAATGTATCCTAATA		
Real-time PCR	*Plasmodium sp.*	Plasmo 1	GTTAAGGGAGTGAAGACGATCAGA		[35]
		Plasmo 2	AACCCAAAGACTTTGATTTCTCATAA		
	*P. falciparum*	P. fal-probe	**FAM**-AGCAATCTAAAAGTCACCTCGAAAGATGACT-**TAMRA**		
	*P. vivax*	P. v-probe	**HEX**-AGCAATCTAAGAATAAACTCCGAAGAGAAAATTCT-**TAMRA**		
	*P. malariae*	P. m-probe	**FAM**-CTATCTAAAAGAAACACTCAT-**MGB**		
	*P. ovale*	P. o-probe	**HEX**-CGAAAGGAATTTTCTTATT-**MGB**		
	*P. ovale*	POF	ATAAACTATGCCGACTAGGTT		[42]
		POR	ACTTTGATTTCTCATAAGGTACT		
	*P. ovale curtisi*	POC-probe	**FAM**-TTCCTTTCGGGGAAATTTCTTAGA-**BHQ1**		
	*P. ovale wallikeri*	POW-probe	**HEX**-AATTCCTTTTGGAAATTTCTTAGATTG-**BHQ1**		

### Nested PCR assay

The above samples were further subjected to nested PCR. For nested PCR, the classical primers targeting the *Plasmodium* 18S small subunit ribosomal RNA (18S ssrRNA) gene were synthesized and used as previously described [43, 44] (the primers are listed in Table 1). The reaction system for the primary round contained 12.5 μl 2 × NovoStar Green PCR Mix (400 μM deoxynucleoside triphosphate [dNTP], 50 U/ml NovoStar Taq DNA polymerase, 4 mM Mg^2+^ and 2 × PCR buffer), 1 μl of each primer (rPLU1 and rPLU5 for 18S *Plasmodium* species, 10 μM) and 1 μl DNA template in a final total volume of 25 μl supplemented with ultrapure water. The amplification was carried out using the following conditions: initial denaturation at 95 °C for 3 min, 30 repeated cycles at 95 °C for 30 s, 55 °C for 1 min, 72 °C for 1 min, followed by a final extension at 72 °C for 5 min. A 1670 bp PCR product was obtained from each sample after the primary amplification, which was then used as the DNA template for the secondary amplification. The reaction system and conditions in the second round were similar to the first round with minor modifications. Briefly, the annealing temperature was adjusted to 56 °C, and the extension time was changed to 30 s, considering the amplification products were shorter in the second reaction. All the products from the secondary amplification were subjected to 1% agarose gel electrophoresis and judged by their band sizes. Products of the nested PCR were used for sequencing if divergent results occurred among the microscopy and the two molecular methods.

### Data analysis

To calculate the diagnostic sensitivity, specificity, positive predictive value (PPV), negative predictive value (NPV), disease prevalence (DP) and accuracy of four *Plasmodium* species, results from nested PCR were considered as the standard. Accuracy was calculated with MedCalc-Diagnostic test evaluation. The remaining five parameters mentioned above and their 95% confidence interval (95% CI) were obtained from online calculator named vassarstats [45]. Briefly, the case number shown positive by both nested PCR and microscopy/real-time PCR was defined as True Positive (a); the case number shown positive by nested PCR but negative by microscopy/real-time PCR was defined as False Negative (b); the case number shown positive by microscopy/real-time PCR but negative by nested PCR was defined as False Positive (c); The case number shown negative by both nested PCR and microscopy/real-time PCR was defined as True Negative (d). Sensitivity = a/ (a + b); Specificity = d/ (c + d); Prevalence = (a + b)/(a + b+c + d); PPV = (Sensitivity × Prevalence)/[(Sensitivity × Prevalence) + (1−Specificity) × (1−Prevalence)]; NPV = Specificity × (1−Prevalence)/[(1−Sensitivity)] × Prevalence + Specificity × (1−Prevalence)]; Accuracy = Sensitivity × Prevalence + Specificity × (1−Prevalence). Flowchart and patients distribution map were drown in Microsoft Office Visio 2010. Other figures were finished in Graphpad Prism 5.0. The trend analysis was carried out by trend estimation in linear regression analysis of SPSS 22.0.

## Results

### Microscopy and RDT detection

**Fig. 1:**
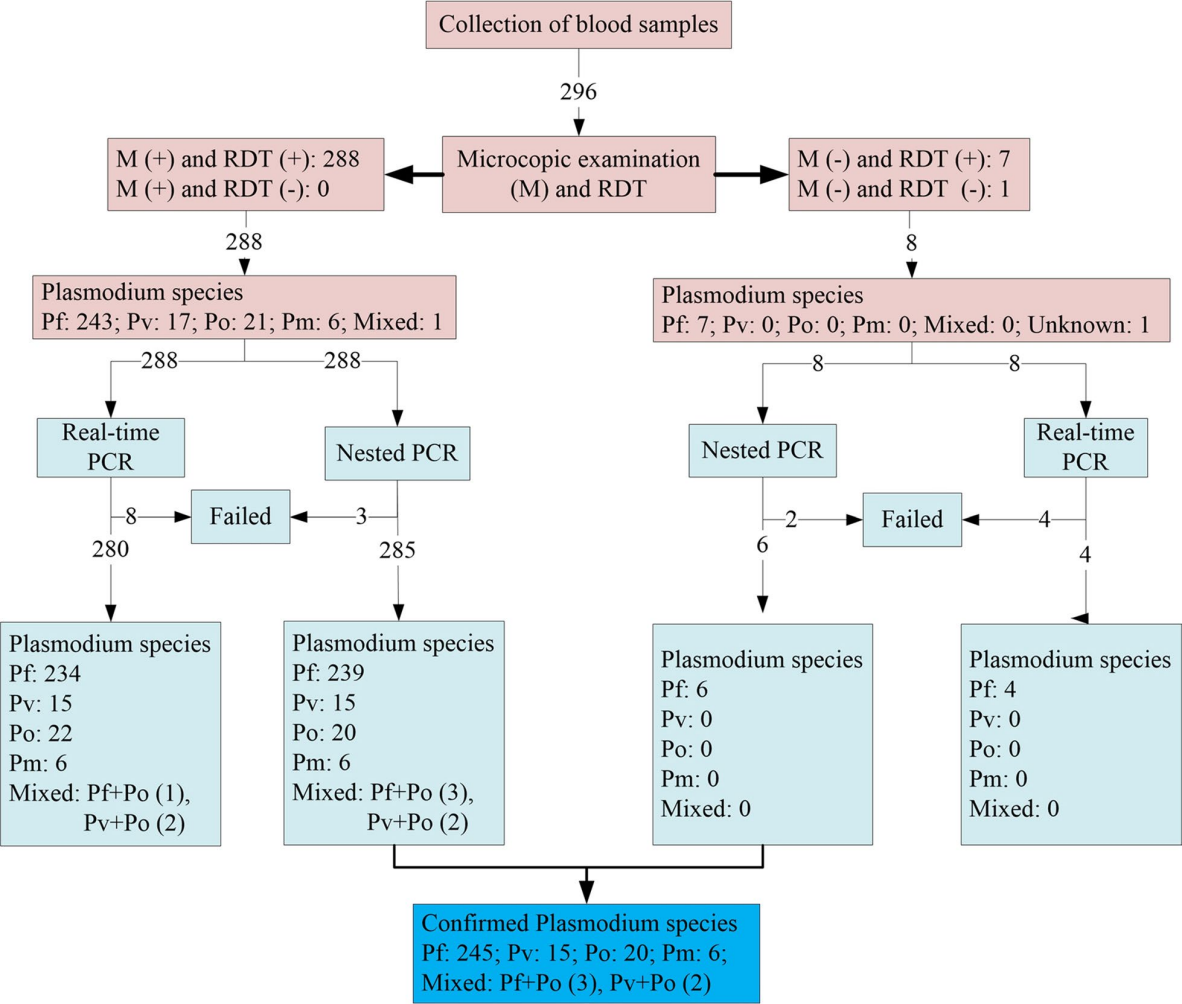
Flowchart detailing the study participation and compliance in Wuhan, China

A total of 296 blood samples from malaria suspected patients were collected (Fig. 1). By using microscopy and RDT, 288 positive samples including 243 *P. falciparum*, 17 *P. vivax*, 21 *P. ovale*, 6 *P. malariae* and 1 coinfection (*P. falciparum *+* P. ovale*) were observed. Seven out of the remaining 8 cases (296 minus 288) were microscopy negative but RDT positive (Fig. 1). The final case was both microscopy and RDT negative but was a suspected patient with clinical symptoms of malaria.

**Fig. 2:**
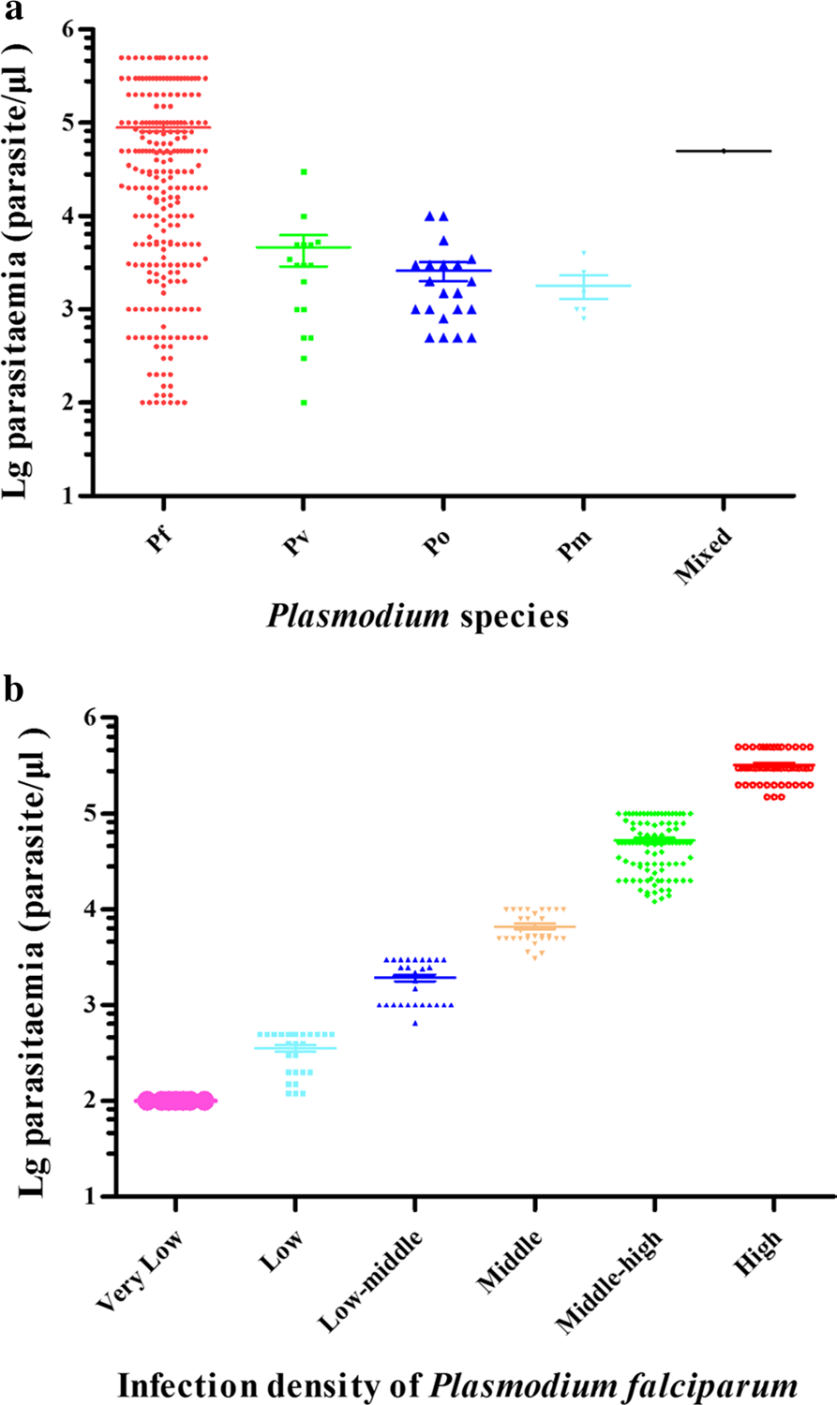
Parasitaemia for *Plasmodium* species infection from imported malaria clinical cases in Wuhan, China. **a** Parasitaemia for four Plasmodium species infection. **b** Infection density of *Plasmodium falciparum*

For the 243 microscopy-positive *P. falciparum* cases (Fig. 2a), parasitaemia ranged from 100 to 500,000 parasite/μl, and the mean parasite density was 88,879 parasite/μl (95% CI 71,794–105,963). These cases were divided into six groups according to their densities: Very Low (≤ 100 parasite/μl), Low (101–500 parasite/μl), Low-middle (501–3000 parasite/μl), Middle (3001–10,000 parasite/μl), Middle-high (10,001–100,000 parasite/μl) and High (> 100,000 parasite/μl). From the very low group to the high group (see Fig. 2b and Table 2), case numbers of *P. falciparum* for each density interval were 7 (2.88%), 26 (10.70%), 34 (33 + 1, 13.99%), 30 (12.35%), 96 (39.51%) and 50 (20.58%), respectively. For *P. vivax*, parasitaemia ranged from 100 to 30,000 parasite/μl and the mean parasite density was 4600 parasite/μl (95% CI 989–8211). For *P. ovale*, parasitaemia ranged from 500 to 10,000 parasite/μl and the mean parasite density was 2610 parasite/μl (95% CI 1348–3871). Of which, 23.53% (4/17) of *P. vivax* and 19.05% (4/21) of *P. ovale* had parasite densities ≤ 500 parasite/μl. All of the 6 *P. malariae* cases had parasite densities from 800 to 4000 parasite/μl and the one mixed infection was 50,000 parasite/μl (see Fig. 2a).

**Table 2:** Determination of *Plasmodium* species by microscopy examination and molecular diagnosis

Microcopic	Parasitaemia (No. of parasites/μl)			Nested PCR^a^		Real-time PCR	
	Species	Intensity (parasite/ul)	No.	Species	No.	Species	No.
Microcopy positive	*P. falciparum*	Very Low (≤ 100)	7	Pf	6	Pf	5
		Low (101–500)	26	Pf	25	Pf	21
		Low-middle (501–3000)	33	Pf	32	Pf	32
		Middle (3001–10,000)	30	Pf	30	Pf	30
		Middle-high (10,001–100,000)	96	Pf	96	Pf	96
		High (> 100,000)	50	Pf	50	Pf	50
		Error correction (3000)	1	Pf + Po	1	Pf + Po	1
	*P. vivax*	From 100 to 10,000	15	Pv	15	Pv	15
		Error correction	2	Pv + Po	2	Pv + Po	2
	*P. ovale*	From 100 to 10,000	19	Po	19	Po	21
		Error correction	2	Pf^a^ +Po	2	Pf +Po	0
	*P. malariae*	From 100 to 10,000	6	Pm	6	Pm	6
	*P. falciparum *+ *P. ovale*	50,000	1	Po	1	Po	1
Microcopy negative	*P. falciparum*^b^		7	Pf	5	Pf	3
	Suspected case		1	Pf	1	Pf	1
Total			296		291		284

^a^The nested PCR products of these cases have been confirmed by DNA sequencing. ^b^ These cases were RDT positive for Pf

### Confirmation of the parasite species with PCR-based methods

These samples were confirmed via both nested PCR and real-time PCR. After reconfirmation by nested PCR (the primers are shown in Table 1), 1.04% (3/288) microscopy-positive and 25% (2/8) microscopy-negative cases failed to be detected. However, 2.78% (8/288) microscopy-positive and 50% (4/8) microscopy-negative subjects were negative by real-time PCR (Fig. 1). Nested PCR products of the 7 cases (12 minus 5), which were *P. falciparum* positive by nested PCR, but negative by real-time PCR, were sequenced, and the results proved they were all *P. falciparum* infections. As shown in Table 2, the 5 negative cases by nested PCR were distributed either in the three groups with relatively low *P. falciparum* density (Very low, Low, Low-middle) or in the microscopy-negative group. For the 12 cases that were not detected by real-time PCR, they were all caused by *P. falciparum.* Their parasite density distributions were highly in accordance with those in the nested PCR detection, including 2 cases in the Very low group, 5 cases in the Low group, 1 case in the Low-middle group and 4 cases in the microscopy-negative group. Additionally, both molecular tools revealed one *P. falciparum* plus *P. ovale* mixed infection case among the microscopy-positive *P. falciparum* samples. The one suspected case negative by both microscopy and RDT was proven to be *P. falciparum* infection.

Of the 17 microscopy-positive *P. vivax* cases, two actually proved to be *P. vivax* plus *P. ovale* mixed infection by both nested and real-time PCR. All 21 microscopy-positive *P. ovale* cases were diagnosed as positive by real-time PCR, but two of them proved to be *P. falciparum* plus *P. ovale* mixed infection by nested PCR and subsequent sequencing. The identification of the 6 *P. malariae* cases showed the same results by three methods. There was also a case that was diagnosed as *P. falciparum* plus *P. ovale* infection by microscopy. However, both nested PCR and real-time PCR revealed the existence of *P. ovale* but not *P. falciparum* (Table 2). Subspecies identification of the 20 single *P. ovale* positive cases showed that they consisted of 10 *P. o. curtisi*, 9 *P. o. wallikeri* and 1 mixture of the two subspecies according to the nested PCR and sequencing. Real-time PCR revealed similar results except for 3 *P. o. curtisi* negative cases.

### Diagnostic profile of *Plasmodium* species by the different methods

As shown in Fig. 1 and Table 3, amongst the 296 samples, there were 243 (82.09%) *P. falciparum*, 17 (5.74%) *P. vivax*, 21 (7.09%) *P. ovale*, 6 (2.03%) *P. malariae*, 1 (0.34%) mixed infection (*P. falciparum *+* P. ovale*) and 8 (2.70%) negative cases according to microscopic observations. Based on the nested PCR, 245 (82.77%), 15 (5.07%), 20 (6.76%) and 6 (2.03%) cases were identified as single infection by *P. falciparum*, *P. vivax*, *P. ovale* and *P. malariae*, respectively, whereas dual species mixed infections included 3 (1.01%) cases of *P. falciparum* + *P. ovale*, 2 (0.68%) cases of *P. vivax* + *P. ovale* and 5 (1.69%) negative cases. Real-time PCR revealed a few minor discrepant results, including 238 (80.41%) *P. falciparum*, 15 (5.07%) *P. vivax*, 22 (7.43%) *P. ovale*, 6 (2.03%) *P. malariae*, 1 (0.34%) mixed infections of *P. falciparum* + *P. ovale*, 2 (0.68%) cases of *P. vivax *+* P. ovale* and 12 (4.05%) negative cases. No triple or quadruple infection were detected in these samples.

**Table 3:** Comparison analysis of diagnostic tools for imported *Plasmodium* species infection in Wuhan, China

	Nested PCR							
Species	*P. falciparum*	*P. vivax*	*P. ovale*	*P. malariae*	*P. falciparum *+ *P. ovale*	*P. vivax *+ *P. ovale*	Negative	Total (%)
Microscopy								
*P. falciparum*	239	0	0	0	1	0	3	243 (82.09)
*P. vivax*	0	15	0	0	0	2	0	17 (5.74)
*P. ovale*	0	0	19	0	2	0	0	21 (7.09)
*P. malariae*	0	0	0	6	0	0	0	6 (2.03)
*P. falciparum* +*P. ovale*	0	0	1	0	0	0	0	1 (0.34)
Negative^a^	6	0	0	0	0	0	2	8 (2.70)
Total (%)	245 (82.77)	15 (5.07)	20 (6.76)	6 (2.03)	3 (1.01)	2 (0.68)	5 (1.69)	296 (100.00)
Real-time PCR								
*P. falciparum*	238	0	0	0	0	0	0	238 (80.41)
*P. vivax*	0	15	0	0	0	0	0	15 (5.07)
*P. ovale*	0	0	20	0	2	0	0	22 (7.43)
*P. malariae*	0	0	0	6	0	0	0	6 (2.03)
*P. falciparum* +*P. ovale*	0	0	0	0	1	0	0	1 (0.34)
*P. vivax *+ *P. ovale*	0	0	0	0	0	2	0	2 (0.68)
Negative	7	0	0	0	0	0	5	12 (4.05)
Total (%)	245 (82.77)	15 (5.07)	20 (6.76)	6 (2.03)	3 (1.01)	2 (0.68)	5 (1.69)	296 (100.00)

^a^ These included 7 RDT positive and 1 RDT negative but suspected cases

### Diagnostic performance of microscopy and real-time PCR compared to nested PCR

Nested PCR was appointed as a reference for the diagnostic summarizing of the *Plasmodium* species as well as the following performance assessment of the diagnostic methods. As shown in Table 4, for microscopy, the sensitivities of identification of *P. falciparum*, *P. vivax*, *P. ovale* and *P. malariae* were 96.77%, 100%, 88.00% and 100.00%, respectively. Specificities for *P. falciparum* diagnosis were 91.67% and 100.00% for the other three species. The probabilities of identification of *P. falciparum*, *P. vivax*, *P. ovale* and *P. malariae* by microscopy from the predicted positive (PPV) and not identification from the predictive negative (NPV) samples were 98.36% and 84.62% (*P. falciparum*), 100.00% and 100.00% (*P. vivax*), 100.00% and 98.91% (*P. ovale*), 100.00% and 100.00% (*P. malariae*), respectively. For the detection of *P. falciparum* by real-time PCR, sensitivity, specificity, PPV and NPV were 96.37%, 100%, 100% and 84.21%, respectively. These four assessment indexes were all 100% for the other three species diagnosed by real-time PCR.

**Table 4:** Performance of microscopy and real-time PCR compared to the reference nested PCR, including sensitivity, specificity, positive predictive value (PPV), negative predictive value (NPV), and disease prevalence (DP)

Nested PCR ™ as standard (confirmed by sequencing)									
Methodological evaluation		Plasmodium species							
		*P. falciparum*		*P. vivax*		*P. ovale*		*P. malariae*	
Nested PCR VS microscopy	Parameters	Positive	Negative	Positive	Negative	Positive	Negative	Positive	Negative
	Positive	240	4	17	0	22	0	6	0
	Negative	8	44	0	279	3	271	0	290
	Analyze	Percentage (95% CI)		Percentage (95% CI)		Percentage (95% CI)		Percentage (95% CI)	
	Sensitivity	96.77 (93.51–98.49)		100.00 (77.08–100.00)		88.00 (67.66–96.85)		100.00 (51.68–100.00)	
	Specificity	91.67 (79.13–97.30)		100.00 (98.31–100.00)		100.00 (98.26–100.00)		100.00 (98.37–100.00)	
	PPV	98.36 (95.58–99.47)		100.00 (77.08–100.00)		100.00 (81.50–100.00)		100.00 (51.68–100.00)	
	NPV	84.62 (71.37–92.66)		100.00 (98.31–100.00)		98.91 (96.57–99.72)		100.00 (98.37–100.00)	
	DP	83.78 (78.97–87.69)		5.74 (3.49–9.21)		8.45 (5.65–12.36)		2.03 (0.83–4.58)	
	Accurancy	95.95 (93.03–97.89)		100 (98.76–100.00)		98.99 (97.07–99.79)		100 (98.76–100)	

Nested PCR VS real time PCR	Parameters	Positive	Negative	Positive	Negative	Positive	Negative	Positive	Negative
	Positive	239	0	17	0	25	0	6	0
	Negative	9	48	0	279	0	271	0	290
	Analyze	Percentage (95% CI)		Percentage (95% CI)		Percentage (95% CI)		Percentage (95% CI)	
	Sensitivity	96.37 (92.99–98.22)		100.00 (77.08–100.00)		100.00 (83.42–100.00)		100.00 (51.68–100.00)	
	Specificity	100 (90.77–100)		100.00 (98.31–100.00)		100.00 (98.26–100.00)		100.00 (98.37–100.00)	
	PPV	100 (98.03–100)		100.00 (77.08–100.00)		100.00 (83.42–100.00)		100.00 (51.68–100.00)	
	NPV	84.21 (71.63–92.09)		100.00 (98.31–100.00)		100.00 (98.26–100.00)		100.00 (98.37–100.00)	
	DP	83.78 (78.97–87.69)		5.74 (3.49–9.21)		8.45 (5.65–12.36)		2.03 (0.83–4.58)	
	Accurancy	96.96 (94.31–98.60)		100 (98.76–100)		100 (98.76–100)		100 (98.76–100)	

The results also revealed that *P. falciparum* (83.78%) infection was the most prevalent, followed by *P. ovale* (8.45%), *P. vivax* (5.74%) and *P. malariae* (2.03%) among the imported malaria cases in Wuhan, China. Since RDT could only distinguish falciparum and non-falciparum *Plasmodium* species infection, RDT was not involved in the methodological evaluation of these samples.

### Origin and years distribution of the patients

**Fig. 3:**
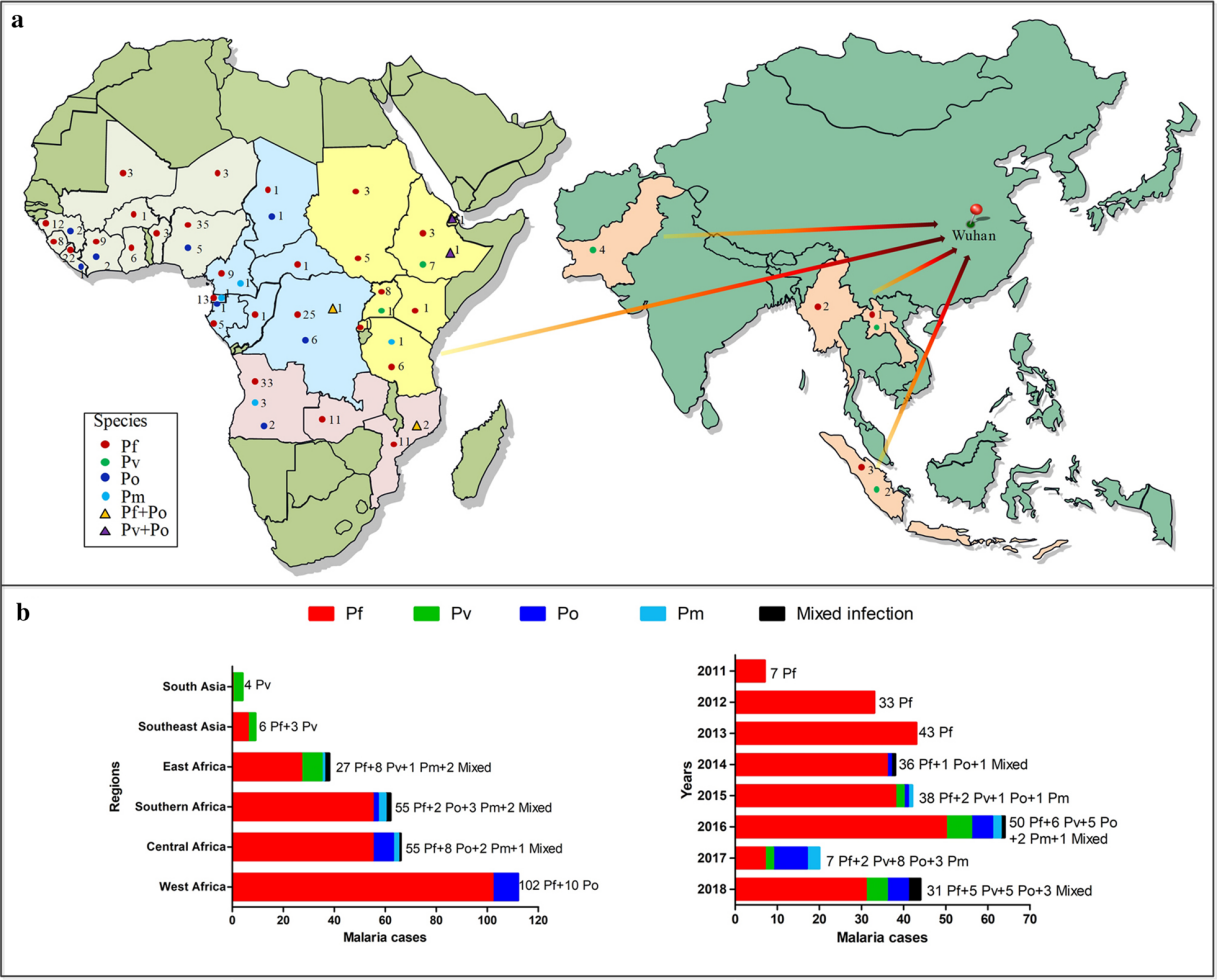
The importation origin and year distribution of imported malaria cases in Wuhan, China, from 2011 to 2018. **a** The map of import`ation origin and cases numbers for the four *Plasmodium* species by country. **b** The number counting of patients infected with each *Plasmodium* species by four importation regions in Africa, two in Asia and years

Tracing the origin of the imported malaria patients demonstrated the patients returned from 28 countries of Africa and 4 countries of Asia (Fig. 3a). Of the total 291 patients confirmed by nested PCR, 112 (38.49%) malaria patients were infected in West Africa, followed by Central Africa (66 cases, 22.68%), Southern Africa (62 cases, 21.31%), East Africa (38 cases, 13.06%), SE Asia (9 cases, 3.09%) and South Asia (4 cases, 1.37%) (Fig. 3b). The distribution of malaria patients caused by *P. falciparum* was in strong accordance with above general tendency, including 102 cases from West Africa, 55 from Central Africa, 55 from Southern Africa, 27 from East Africa and 6 from SE Asia. Amongst the *P. vivax* patients, 8 returned from East Africa. The remainders all returned from SE Asia (3 cases) and South Asia (4 cases), respectively. The *P. ovale* patients returned from West Africa (10/20), Central Africa (8/20) and Southern Africa (2/20). Only 6 *P. malariae* infected patients were involved, and three returned from Southern Africa, two from Central Africa and one from East Africa. The five mixed infection patients also returned from the same three regions as the *P. malariae* infection group.

From 2011 to 2016 (Fig. 3b), the number of imported malaria patients has obviously increased yearly (F = 15.11, P = 0.018). Luckily, only 20 imported malaria patients were reported by the CDC and hospitals of Wuhan in 2017. However, the number of clinical malaria patients in 2018 increased to 44 cases. It is interesting to note that the predominant species was *P. falciparum* throughout the surveyed period. However, it is worth mentioning no consistent increasing trends in the prevalence of *P. falciparum* parasites infection (F = 0.078, P = 0.790) was detected yearly. Other *Plasmodium* species, including *P. vivax*, *P. ovale* and *P. malariae* single infection or mixed infection, were not identified until 2014. In the next 5 years (2014–2018), patients infected with *P. vivax* (15 cases), *P. ovale* (20 cases), *P. malariae* (6 cases) and mixed parasite species (5 cases) were seen. In this period (2014–2018), the total imported non-falciparum cases showed no increasing trend in prevalence by year (F = 4.765, P = 0.117). Neither *P. vivax* (F = 0.217, P = 0.687) nor *P. ovale* (F = 5.000, P = 0.111) significantly increased by year.

## Discussion

In 2020, China hopes that national malaria elimination will become a real possibility based on effective control measures [4]. Although no indigenous malaria case has been reported in China since 2017, imported malaria is becoming a serious obstacle to malaria elimination. If China plans to achieve the stated goals on schedule, continuous surveillance of imported malaria is an essential measure. As an important city in Central China, Wuhan has a population of 11.08 million. It also faces similar problems: imported malaria cases increased annually accompanied by the disappearance of indigenous malaria cases since 2013 [5]. Thus, it is necessary to monitor the epidemic status of imported malaria in China, including in Wuhan. Furthermore, identification of the malaria parasite species with timely and effective methods could provide valuable information for developing suitable clinical treatment.

In the current study, several discrepant results were obtained by the four different diagnostic methods, which ultimately revised 5 cases from single to mixed infection and 6 cases from negative to positive. The confirmed data revealed that 95.5% of the imported malaria patients had returned from Africa, and *P. falciparum* was the dominant imported species. Analysis of the performance of the diagnostic methods involved also revealed some limitations in their usage. Of the 243 *P. falciparum* cases identified by microscopy, one case later proved to be infected with *P. falciparum* plus *P. ovale* by both molecular tests. Two microscopy-positive *P. vivax* cases were also found to be cases of *P. vivax* plus *P. ovale.* Likewise, two patients with single *P. ovale* infection by microscopy were proven as *P. ovale* plus *P. falciparum* infection by nested PCR. In previous studies, *P. ovale* was also frequently reported to be involved in mixed infection with other *Plasmodium* species [20, 46, 47]. These results demonstrate the limitations of microscopy in the identification of mixed infection and the highly specialized requirements of the trained observers [48]. The identification of *P. o. curtisi* and *P. o. wallikeri* in 20 single *P. ovale* infection cases, which were unable to be distinguished by microscopy, also highlighted the advantages of molecular tools in species identification. Their diagnosis will offer a clue for the precise treatment of malaria patients. Subsequently, nested PCR also proved 6 cases as *P. falciparum* infection, which included 5 microscopy-negative but RDT-positive cases and one double-negative case. This was probably because the level of parasitaemia was too low to be observed in these cases. As reported in previous studies [49, 50], microscopy has a level of detection about 50–500 parasites/μl for *Plasmodium*, but the threshold, 50 parasites/μl, is rarely noticeable unless under optimum conditions. This means that it is quite difficult for observers to accurately identify the species with relatively low parasitaemia, especially in mixed infections.

In the analysis of sensitivity, specificity, PPV and NPV, nested PCR-based detection was chosen as the reference standard. Actually, nested PCR is commonly selected as a reference method [51, 52]. By comparing to nested PCR, microscopy (96.77%) showed similar sensitivity to real-time PCR (96.37%) for *P. falciparum* identification. However, the specificity of microscopy was only 91.67%, lower than the 100% of rea1-time PCR detection. By designing complementary probes to target gene sequences, the Taqman probe is another guarantee besides the primers to enhance the specificity and avoid false-positive results [38]. The NPV for *P. falciparum* detection was approximately 85% in both microscopy and real-time PCR detection, indicating a relatively high false-negative rate of approximately 15% for these two methods. A false-negative rate as high as 19.4% for microscopy was reported in a previous study [53]. In another study, NPV was as low as 65.8% for microscopy detection [54]. These indicated the persistent limitations and instability of microscopy diagnosis. For real-time PCR in the present study, its high false-negative rate was mainly attributed to its failure to diagnose 9 *P. falciparum* cases consisting of 7 samples with low parasite density (0–500 parasites/μl) and 2 mixed infection samples. In a previous study, the same real-time PCR primers and probes were assessed [55]. Although only 198 of 207 (95.7%) malaria samples were accurately diagnosed at the species level, the NPV for *P. falciparum* detection was 100%. This indicated that diagnostic performance may vary with the reaction conditions, including the DNA quality, reaction system and even the performance of the laboratory technicians, remaining us of the significance for optimization of the reaction system. For *P. ovale*, the sensitivity (88.00%) of microscopic identification was relatively low, and the NPV for *P. ovale* was 98.91%. Two of the 3 *P. ovale* cases that failed to be detected by microscopy were proved to be *P. ovale* plus *P. vivax* mixed infections. This may be due to the similar morphology between *P. ovale* and *P. vivax* [12]; as a result, the species with a relatively lower parasitaemia level may be hidden. The four indexes (Table 4) in real-time PCR detection were all 100% for *P. vivax*, *P. malariae* and *P. ovale* detection, revealing the high consistency between the two molecular tools in the identification of these three species. *Plasmodium malariae* could be accurately diagnosed by all three methods. This may be because only 6 *P. malariae* cases were involved in the study. The primers for real-time PCR used in this study have been used in several previous studies [35, 56]. However, it has been reported that simultaneous amplification of different templates was difficult and that the primers preferred to amplify species with higher parasitaemia as a result of competition for the shared primers (Plasmo1 and Plasmo2). In the present data, 7 cases out of the total 9 nested PCR-positive patients failed to be identified by real-time PCR. Actually, all had low parasitaemia and the other two had mixed infections. Similar phenomena were also reported in previous studies [57, 58]. A false negative result means a person with *Plasmodium* infection failed to be diagnosed. This could cause a misdiagnosis, delayed treatment of malaria and even lead to a life-threatening problem, especially for *P. falciparum* infection. Beyond the individual consequences, false-negative patients may also spread malaria and produce resistance to anti-malarial drugs if given incorrect treatment due to the lack of an exact diagnosis [53].

Although nested PCR showed the highest sensitivity and real-time PCR had the best specificity, the role of microscopy and RDT is not controversial. Use of the two molecular methods relies on DNA extraction from blood samples. If errors happen during the extraction, a false-negative would probably occur. For example, in a previous study, 10 malaria cases failed to be identified by molecular methods due to problems with the nucleic acid extraction, and the reasons were only identified after repeated experiments [59]. Therefore, the combined use of the four methods is quite necessary and highly recommended if conditions permit. Both microscopy and RDT can offer a preliminary result and suggest further diagnostic directions. Based on this valuable information, the *Plasmodium* species can then be confirmed by molecular methods, including nested PCR and real-time PCR.

The origin of infection of the patients was consistent with a previous report referring to the epidemiologic features of imported malaria in China [60]. In the epidemic regions of Africa and SE Asia, the natural environment, healthcare and situation of economic development may have primary influences on malaria infection. Chinese people overseas, especially those that do not live for long periods in epidemic areas, usually lack adaptive immunity and related awareness of local *Plasmodium* infection [60]. Additionally, there are frequently asymptomatic patients [61]. Workers returned from these areas, particularly West Africa, Central Africa, and Southern Africa, should be encouraged to undergo malaria testing. As the dominant malaria species, the prevalence and origins of *P. falciparum* were similar to other studies [53, 62]. Therefore, prevention and treatment against *P. falciparum* should be highly considered in Wuhan. Over the years (from 2014 to 2018), the *Plasmodium* species leading to imported malaria were becoming diversified, just as in other provinces of China, including Shandong [59], Zhejiang [62], Shanxi [63] and Chongqing municipality [64]. In this period, the imported non-falciparum cases and *P. vivax*/*P. ovale* cases showed no significantly increasing trend by year, but more attention should be focused on this increasing *Plasmodium* species diversity. The increase in imported *P. vivax* and *P. ovale* cases may cause the re-introduction of malaria into regions without cases [65, 66]. *Plasmodium malariae* can survive in humans for many years and keep infectivity of its vector at a truly low parasitaemia load [67]. The sharp decrease in imported malaria cases in 2017 may be a good sign, indicating the successful performance of malaria control. However, the cases increased again in 2018, warning us about the challenges of complete elimination of *Plasmodium* infection worldwide, even in China.

Additionally, results in the present study showed no significant seasonal correlation (P = 0.759) by the Pearson correlation analysis among the imported malaria cases during the study period. One small peak of the seasonal index [68] appeared in May (1.44) to June (1.32). Another study of malaria in Hubei province from 2005 to 2016 revealed that indigenous malaria had an obvious seasonal fluctuation during their study period, with a peak of seasonal index in August (2.26). However, only three small peaks of seasonal index (all approximately 1.3) for imported cases were found, including one in January, a second one in May and the last one in September [5]. This indicated that there are differences in the incidence of monthly cases between indigenous and imported malaria. Comparisons with five neighbouring provinces/cities of Hubei also showed that no obvious correlation existed between season and imported malaria cases in these regions [64, 69–72]. Even so, two time slots should be paid more attention for the prevention of imported malaria. One is the Chinese Spring Festival, which leads to the most frequent migration between provinces in China and brings many overseas Chinese home [5, 63, 64, 71]. Another is the anopheles active phase [5, 64, 70–72]. Importantly, commuting and the movement of imported malaria cases between provinces should also be of concern, which has been reported in another city of China [71].

Indeed, besides an early and precise diagnosis for proper treatment, some preventative measures should also be emphasized from two major aspects before people leave for an epidemic area: (1) Prevention of mosquito bites. Travellers could protect themselves by using insect repellents, insecticide-treated nets, aerosol sprays and protective clothing. (2) Proper usage of chemoprophylactic anti-malarial drugs. Anti-malarial prophylactic regimens may not provide complete protection, but proper chemoprophylaxis could significantly lower the risk of fatal malaria. According to the prevalence of *Plasmodium* species at the destination and the conditions of the travellers (healthy adults, children, pregnant women or immunosuppressed travellers), different chemoprophylactic schedules should be advised by the local government according to the guide on international travel and health published by the WHO. Only a combination of prevention and treatment can lead to the achievement of the goal of malaria elimination around the world.

## Conclusions

The efficiency of 18S ssrRNA-based nested PCR and real-time PCR in the differentiation of four *Plasmodium* species and two subspecies of *P. ovale* was assessed. The results highlighted, once again, that PCR-based methods are irreplaceable for exact species determination of imported malaria cases, especially for mixed infections. Therefore, the combined use of the four methods should be emphasized, if conditions permit. These data are a very important epidemiological resource and could supply guidance for the surveillance, prevention and timely treatment of imported malaria in Wuhan, China. The guiding effect is also significant for epidemic areas, as shown in the origin distribution survey, and for countries at potential high risk of importing malaria worldwide.

## Data Availability

The datasets analysed in this study are available from the corresponding author on reasonable request.

## Abbreviations

RDT: Rapid diagnosis test
PPV: Positive Predictive value
NPV: Negative Predictive value
DP: Disease prevalence
CDC: Center for Disease Prevention and Control
